# Exploring the perspectives of pharmaceutical experts and healthcare practitioners on senolytic drugs for vascular aging-related disorder: a qualitative study

**DOI:** 10.3389/fphar.2023.1254470

**Published:** 2023-10-06

**Authors:** Li Ping Wong, Haridah Alias, Kit Mun Tan, Pooi Fong Wong, Dharmani Devi Murugan, Zhijian Hu, Yulan Lin

**Affiliations:** ^1^ Centre for Epidemiology and Evidence-Based Practice, Department of Social and Preventive Medicine, Faculty of Medicine, Universiti Malaya, Kuala Lumpur, Malaysia; ^2^ Department of Epidemiology and Health Statistics, The School of Public Health, Fujian Medical University, Fuzhou, Fujian, China; ^3^ Department of Medicine, Faculty of Medicine, Universiti Malaya, Kuala Lumpur, Malaysia; ^4^ Department of Pharmacology, Faculty of Medicine, Universiti Malaya, Kuala Lumpur, Malaysia

**Keywords:** senolytics, vascular aging, premature aging, vascular aging drugs, senotherapeutics

## Abstract

**Objective:** The field of targeting cellular senescence with drug candidates to address age-related comorbidities has witnessed a notable surge of interest and research and development. This study aimed to gather valuable insights from pharmaceutical experts and healthcare practitioners regarding the potential and challenges of translating senolytic drugs for treatment of vascular aging-related disorders.

**Methods:** This study employed a qualitative approach by conducting in-depth interviews with healthcare practitioners and pharmaceutical experts. Participants were selected through purposeful sampling. Thematic analysis was used to identify themes from the interview transcripts.

**Results:** A total of six individuals were interviewed, with three being pharmaceutical experts and the remaining three healthcare practitioners. The significant global burden of cardiovascular diseases presents a potentially large market size that offer an opportunity for the development and marketability of novel senolytic drugs. The pharmaceutical sector demonstrates a positive inclination towards the commercialization of new senolytic drugs targeting vascular aging-related disorders. However potential important concerns have been raised, and these include increasing specificity toward senescent cells to prevent off-site targeting, thus ensuring the safety and efficacy of these drugs. In addition, novel senolytic therapy for vascular aging-related disorders may encounter competition from existing drugs that treat or manage risk factors of cardiovascular diseases. Healthcare practitioners are also in favor of recommending the novel senolytic drugs for vascular aging-related disorders but cautioned that its high cost may hinder its acceptance among patients. Besides sharing the same outcome-related concerns as with the pharmaceutical experts, healthcare practitioners anticipated a lack of awareness among the general public regarding the concept of targeting cellular senescence to delay vascular aging-related disorders, and this knowledge gap extends to healthcare practitioner themselves as well.

**Conclusion:** Senolytic therapy for vascular aging-related disorders holds great promise, provided that crucial concerns surrounding its outcomes and commercial hurdles are effectively addressed.

## 1 Introduction

Cellular senescence is defined as a state of irreversible cell cycle arrest that alters cellular characteristics and functions in response to various stress stimuli (Florido et al., 2011). Senescent cells remain metabolically active, but exhibit distinct cellular morphology, mitochondrial dysfunction, disrupted proteostasis, aberrant nutrient sensing as well as expressing senescence markers such as p16, senescence-associated β-galactosidase (SA-β-gal), senescence-associated heterochromatin foci (SAHF) and others (von Zglinicki et al., 2021). Although it is a normal physiological process that halts the proliferation of dysfunctional cells, the accumulation of senescent cells within the tissue microenvironment is detrimental. This is because senescent cells assume the senescence-associated secretory phenotype (SASP), which secretes a plethora of soluble mediators including proinflammatory cytokines, chemokines, matrix metalloproteinases, growth factors and others. SASP factors can induce paracrine senescence of the surrounding cells and chronic low-grade inflammation within the tissue microenvironment, ultimately resulting in tissue damage and dysfunction (Acosta et al., 2013; Gorgoulis et al., 2019). Cellular senescence has been implicated in both the development and progression of age-related disorders such as cardiovascular diseases, diabetes, neurodegenerative disorders, and many others (McHugh and Gil, 2018; Prata et al., 2018). A recent study showed that following myocardial infarction in mice, senescent cardiomyocytes cause pathological cardiac remodelling and promote senescence of other cardiac cell types such as fibroblasts and endothelial cells (Redgrave et al., 2023). The removal of the senescent cells attenuated maladaptive cardiac remodelling and subsequently improved cardiac function (Redgrave et al., 2023). In another study involving human heart tissue, increased number of senescent human cardiac stem cells (hCSCs) was observed in tissue derived from type 2 diabetes mellitus (T2DM) patients compared to the non T2DM group. These T2DM-hCSCs also exhibited a defined SASP profile and had reduced regenerative capacity (Marino et al., 2022). The findings from these and many earlier studies underscore the pivotal role of senescent cell accumulation and SASP in driving the development and progression of age-related diseases.

Of late, researchers have been actively investigating the mechanisms underlying cellular senescence and identifying potential drug candidates or senolytic therapy that could modulate or eliminate senescent cells. The functions of immune system gradually decline with increasing age, thus compromising the clearance of senescent cells. Senolytic agents could play a critical role in selectively inducing apoptosis of senescent cells or enhancing their immune-mediated clearance, thereby reducing age-related senescent cells burden in tissues and organs (Kirkland et al., 2017). Elimination of senescent cells with senolytics has been demonstrated to improve tissue functions and prevent the progression of aging-related diseases. In addition, senomorphics or compounds that can modify the SASP secretome of senescent cells rather than eliminating them have been explored to attenuate chronic inflammation and its deleterious effects (Cuollo et al., 2020). Fine-tuning the SASP secretome can preserve the beneficial aspects of cellular senescence such as promoting wound healing and tumour immune surveillance.

Among all the non-communicable diseases, cardiovascular diseases (CVDs) hold the unfortunate distinction of being the leading cause of death globally, with an estimated number of nearly 18 million people worldwide dying each year (World Health Organization, 2023). Likewise in Asia, CVDs represent an emerging epidemic and are also the leading cause of death (Zhao, 2021). The impact of CVDs in Asia is particularly alarming, with a significant percentage of CVD deaths in Asia classified as premature death. In Asia, the proportion of premature deaths (39%) due to CVDs was notably higher compared to the United States (23%), Europe (22%), and the global average (34%) (Zhao, 2021). The alarming statistic underscores the pressing need for effective CVD prevention measures to address the substantial impact of this disease on global health. This need is especially critical in countries within Asia where the burden of CVDs is particularly high. Consequently, there has been a surge of interest in evaluating senolytic agents as potential treatment for aging-related disorders such as cardiovascular diseases. In recent years, several potential senolytic agents that hold promise in treating age-related diseases, including those targeting senescent cells within the blood vessels have been identified. For instance, a cocktail of dasatinib (D) plus quercetin (Q) was found to have senolytic effect. D + Q could improve the function of the vascular endothelium, reduce the stiffness of blood vessels (Suda et al., 2023) and ameliorate pathological cardiac remodeling and dysfunction in mice following myocardial infarction (Salerno et al., 2022). Another senolytic drug, ABT-263 (navitoclax), has demonstrated the capability to eliminate senescent cardiomyocytes (Song et al., 2020), attenuate adverse cardiac remodeling and improve cardiac function following cardiac ischemia-reperfusion injury in mice (Dookun et al., 2020).

The growing recognition of senescence as a key contributor to aging-related comorbidities and the great potential of senolytic therapy has garnered considerable attention from both academic researchers and biotechnology companies. While the potential availability of pharmaceutical interventions targeting senescence to improve vascular health is a fascinating new avenue, there are concerns regarding the acceptance of senolytic drugs. It is natural for new medications or treatment approaches to face skepticism or hesitation, especially when they are relatively unfamiliar. Skepticism likely results from the fact that aging is not traditionally seen as a disease, but rather a natural and universal process (Kennedy and Pennypacker, 2014). Furthermore, drugs targeting the fundamental aging mechanism such as cellular senescence to treat multiple chronic diseases with shared underlying mechanisms may seem unimaginable to many people. These concerns may not be unfounded as potential challenges and pitfalls in the clinical translation of senolytics have been highlighted (Owens et al., 2021). Furthermore, while the senolytic combination drug D + Q has shown promising safety profiles, there remains some uncertainty regarding their risk-benefit ratio (Wissler Gerdes et al., 2021). Targeting signaling pathways that control SASP factors such as NF-κB, JAK/STAT and MTOR pathways with pharmacological inhibitors seems to be a more feasible approach (Cuollo et al., 2020). However, there remain challenges such as the considerable heterogeneity of the SASP secretome that is dependent on the type of stimulus, cells and environmental conditions. This variability is further complicated by those produced in comorbidities, presenting significant hurdles in identifying senescence specific SASP profile for targeting (Birch and Gil, 2020). This could explain why senolytics such as D + Q and fisetin have transitioned to phase II trials for several age-related diseases in elderly individuals with subsequent trials planned in the pipeline (Wissler Gerdes et al., 2021; Raffaele and Vinciguerra, 2022).

The viewpoints of the pharmaceutical industry regarding the novel senolytic drugs for vascular aging are of considerable importance. The pharmaceutical industry plays a crucial role in bridging the gap between scientific breakthroughs to actual production or manufacturing of drugs and translation to clinical use. As the potential senolytic interventions progress through preclinical and clinical development, the pharmaceutical industry needs to be equipped with expertise and resources to support the translation into approved therapy for its intended use. Gaining insights from the pharmaceutical industry is vital in order to understand their concerns and resource requirements, which in turn can facilitate the transformation of the promising use of senolytic drugs into effective therapies for patients.

Prescribing and recommendation by healthcare practitioners were reported as one of the most powerful predictors of new drug uptake (Lublóy, 2014). Hence, healthcare practitioners play a vital role in promoting and facilitating the utilization of potential new senolytic agents among patients, should they become available in the future. Healthcare practitioners are consistently ranked as one of the most trusted sources of health information (Hong and Oh, 2020). Healthcare practitioners also possess the knowledge and expertise required to educate patients about the mechanisms of action of senolytic agents, as well as the specific conditions of diseases for which senolytic therapy is intended. Exploring the perceptions of healthcare practitioners regarding the benefits and potential barriers associated with recommending senolytic therapy is crucial, as they can significantly enhance patients’ trust and adoption of this therapy. Moreover, identifying the knowledge gaps and uncertainties healthcare practitioners have regarding specific aspects of the new therapy can inform the development of tailored educational materials and training programs. This will equip healthcare practitioners with effective communication strategies when discussing senolytic drugs with patients in the future.

Given the aforementioned concern, the objective of this study is to employ qualitative inquiry to investigate the perspectives of healthcare practitioners and pharmaceutical experts regarding the novel senolytic therapy for vascular aging-related disorders. Specifically, the study aims to gather insights from pharmaceutical experts involved in the prospect of the pharmaceutical industry in bridging the gap between scientific breakthroughs to actual production or manufacturing of senolytic drugs for vascular aging-related disorders. Additionally, healthcare practitioners’ perspectives will be sought to identify the advantages and potential challenges of recommending senolytic therapy to patients.

## 2 Materials and methods

### 2.1 Study design and study instrument

The recruitment of participants utilized purposive sampling, aiming to include individuals with diverse work experiences and positions. Efforts were made to recruit participants who exhibited a wide range of variation in these aspects. The interviews were conducted through one-on-one sessions spanning from May to June 2022. Due to the study period coinciding with the COVID-19 pandemic, the researchers decided to conduct online interviews. The duration of the interviews ranged from 30 to 50 min. All interviews were recorded and transcribed verbatim by an independent transcriber. To ensure accuracy, the transcriptions were verified by another independent researcher. Data analysis occurred simultaneously with the data collection process. Sampling was conducted until data saturation was achieved, which involved repeating previous data to ensure comprehensive coverage.

Thematic analysis was used to identify themes from the interview transcripts. During the data analysis phase, the standards for credibility, dependability, transferability, and conformability were employed to ensure the accuracy and validity of the reporting of findings. Firstly, the researchers thoroughly read the transcripts to gain a comprehensive understanding of the entire content. Once they had familiarized themselves with the data, they proceeded to identify codes. The codes were subsequently grouped based on shared concepts, thereby forming the primary categories. The primary categories were further organized into secondary categories based on the relationships observed between them. The next step involved identifying themes and interpreting the data to derive meaningful insights. Data were managed using Nvivo 10 [Burlington, MA, QSR International (Americas) Inc.] (QSR International Pty Ltd., 2012).

### 2.2 Ethical consideration

This study was approved by the University of Malaya Research Ethics Committee (UM.TNC2/UMREC_1507). All participants voluntarily participated in the study and provided their oral consent. Furthermore, oral consent was also obtained specifically for recording the interviews. The participants were assured of the confidentiality and anonymity of the information they provided.

## 3 Results

**FIGURE 1:**
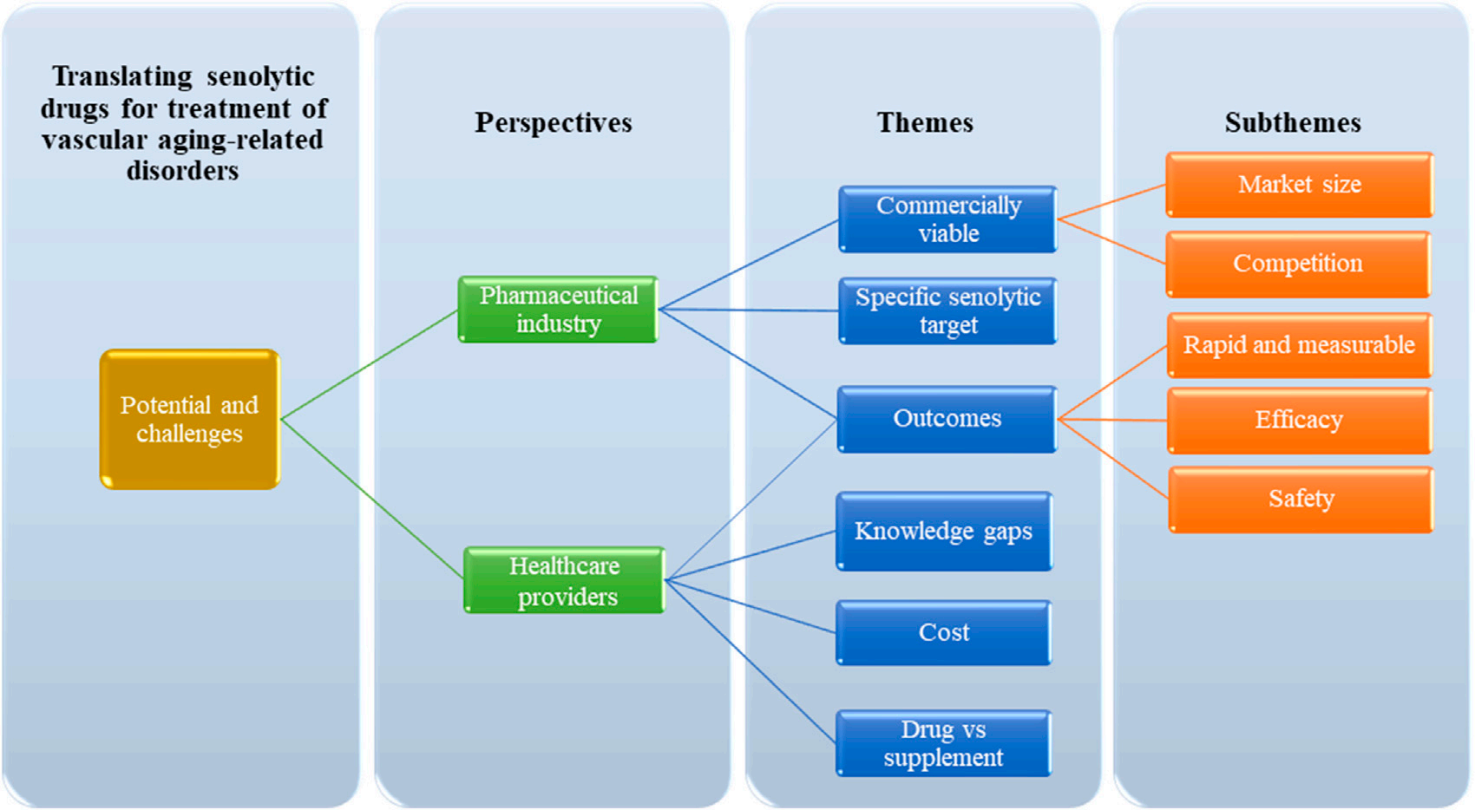
Identified themes in the discovery of potential and challenges associated with the introduction of senolytic therapy.

The study involved six participants, including three pharmaceutical experts and three healthcare practitioners. The study analyses of the theme are focused on identifying the potential and challenges associated with the introduction of senolytic therapy for vascular aging-related disorders across different contexts. The identified themes are presented in Figure 1.

### 3.1 Perspectives from pharmaceutical experts

#### 3.1.1 Commercially viable

The pharmaceutical experts reported that the industry places significant emphasis on developing commercially viable products when translating laboratory findings into production. The consensus among all the pharmaceutical experts is that market size is regarded as a critical factor in determining the viability of a new product. They highlighted the importance of pharmaceutical companies evaluating factors such as the potential patient population and disease prevalence to determine the commercial potential of a product. This evaluation enables companies to prioritize resources and focus on developing products that have a greater likelihood of achieving success in the market.

“There are many promising drugs that never made it to market simply because they lacked commercial viability.”

In addition to considering market size and commercial potential, the pharmaceutical experts highlight the crucial aspect of developing products that specifically target unmet medical needs. This implies focusing on conditions or diseases for which there is a lack of effective treatment options or where existing treatments have limitations.

“There are many anti-hypertensive drugs, which are cheaper, why would one wants a new therapy which may not work?”

The pharmaceutical experts also discussed the potential competition between new senolytic drugs for vascular aging-related disorders and current drugs or supplements targeting blood vessels.

“Why would someone opt for a new therapy, which is totally new and that may not be effective when there are already numerous cheaper anti-hypertensive drugs available?”

“Particularly in the area of aging, there is a wide range of herbal supplements and nutri-supplements.”

#### 3.1.2 Rapid and measurable outcomes

According to pharmaceutical experts, pharmaceutical companies frequently prioritize the commercialization of drugs that demonstrate the ability to yield quick and measurable or quantifiable outcomes. It allows users to assess the drugs’ effectiveness.

“If someone takes the drug and the results can be measured, and in a short time without having to wait for years and not knowing if it works.”

“For senolytic therapy to delay vascular aging, it is good that a measurable indicator can be established, in terms of immediate results or how quickly the drugs take effect.”

#### 3.1.3 Senolytic targets

Pharmaceutical experts spoke about the importance of identifying the senescence induction mechanisms, as well as its pro-survival pathways or possible targets. They recognized that although the identification of therapeutic targets for senolytic drugs can pose challenges for researchers, it brings significant opportunities and potential. Identifying the specific senolytic targets allows pharmaceutical companies to design and develop the right compounds that specifically eliminate targeted senescent cells. Pharmaceutical experts viewed that if a senolytic therapy has a specific target on vascular aging-related disorders, it can lead to increase efficacy, reduces the risk of adverse effects, and improves the safety profile of the therapies. There is an acknowledgment of the importance of the development of senolytic therapies with a specific emphasis on particular disease areas.

“It is crucial to prioritize the development of senolytic therapy for specific diseases such as cancer, cardiovascular diseases, or neurodegenerative diseases. This enables the pharmaceutical industry to target sales and marketing strategies.”

Additionally, the pharmaceutical experts also emphasized the importance of overcoming toxicity hurdles and ensuring efficacy before proceeding with the commercialization process.

### 3.2 Perspectives from healthcare practitioners

#### 3.2.1 Understanding the new senolytic therapeutic paradigm

The concept of senolytic is relatively new and is not taught in medical schools. Therefore, healthcare practitioners noted that they require a deeper understanding of the new therapeutic paradigm before they can confidently recommend it to their patients.

When probed, healthcare practitioners view that they should play a role to educate the patients about the new senolytic drugs if it is available. Healthcare practitioners also anticipated that recommending senolytic therapy to patients or explaining it to them would be relatively easier for individuals with a higher level of education, while those with a lower level of education may have difficulty understanding senotherapeutic concepts.

#### 3.2.2 Outcome and safety profile

Most of the interviewed healthcare practitioners noted that they place great emphasis on the safety profiles of new senolytic drugs when making recommendations to patients, particularly those who are frail. If the new senolytic becomes available, they would require more concrete evidence regarding its safety profile before considering its introduction to their patients.

“If a blood vessel is diseased and there is something that can reduce the chances or delay the progression of the disease, then it is highly beneficial.”

“Most importantly, does it contribute to a better outcome? If there is a drug that prevents the aging of blood vessels, improves circulation and provides noticeable outcomes, it would be of great advantage.”

The second foremost important is the outcome or efficacy of a new senolytic drug for vascular aging.

“In the case of frail elderly individuals who are already on multiple medications, one of the primary concerns when considering the addition of a new senolytic therapy is the possibility of drug-drug interactions. However, if the therapy shows promising outcomes and is also well tolerated, there is a rationale for giving it a try.”

“Usually patients trust doctors very well, so it is important that clinicians ensure the safety of drugs before recommending to patients.”

There is also a concern about the duration of treatment with the medication if the new senolytic drugs become available. Since aging is a gradual process, it would pose a challenge to consistently take the medication over a long period.

#### 3.2.3 Cost

Healthcare practitioners also said that a primary concern raised by patients when clinicians recommend new drugs is the cost. Therefore, it is of utmost importance that despite the new senolytic paradigm represents a breakthrough in treating vascular aging-related disorders, they should be made accessible and affordable for the general population.

“Usually when we recommend new drugs, for example, anti-hypertensive drugs, patients will start to query is it worth. For government servants, they would expect the drug to be given free?”

“If very minimal benefit but the cost is higher, it is cost-effective to patients?”

#### 3.2.4 Drugs versus supplement

A physician also pointed out that as aging is not considered a disease, this can make it extremely difficult to introduce the new senolytic drug to patients. For instance, if it is classified as a supplement, the drug may be more freely available to patients than it would be if it was classified as a pharmaceutical drug. However, even though the new senolytic drug is being presented as a supplement, it still faces its own set of challenges.

“In this government hospital, the medications provided to many patients are free of charge. If the senolytic therapy is classified as a supplement, patients would need to bear the cost themselves. Cost will also be an issue, if the drug is not free, high cost may not be affordable for many patients.”

## 4 Discussion

Health providers and pharmaceutical experts viewed that there are certain challenges and considerations associated with measuring outcomes and assessing the effects of senolytic therapies. One of the primary issues is that measurable outcomes may not be immediate. Senescent cell clearance and subsequent tissue regeneration may take time to manifest noticeable effects. More importantly, to date there are no tools such as imaging probes or biomarkers that can measure the clearance of senescent cells from tissue. The lack of such simple tools not only hamper the identification of senolytic drugs which are truly specific for senescent cells but also for monitoring therapeutic efficacy (Niedernhofer and Robbins, 2018). While senolytic therapies offer promising potential benefits, it is crucial to acknowledge that observable outcomes may not occur immediately. Patients and healthcare practitioners might not observe immediate improvements in vascular health or other age-related conditions following the administration of senolytic drugs.

Clinical trials should focus on investigating the outcomes of senolytic therapy, with an emphasis on the understanding the duration of time required for significant improvements to occur. In terms of patient education, it is recommended to provide clear information about the expected outcomes of senolytic therapy and emphasize that measurable results may take time to manifest. Additionally, promoting a holistic approach to health and wellbeing, including healthy lifestyle choices, can complement the potential benefits of senolytic therapy and contribute to overall wellbeing.

Secondly, the introduction of senolytic drugs for vascular aging-related disorders should include measurable outcomes that indicate improvement in vascular health. One common method for assessing blood vessel dilation is through the use of vascular function tests, such as endothelial function assessments (Patvardhan et al., 2010). Techniques like flow-mediated dilation can be used to evaluate the ability of blood vessels to dilate in response to increased blood flow (Raitakari and Celermajer, 2000). Additionally, techniques such as Doppler ultrasound can be employed to measure blood flow velocity and volume in specific arteries or regions of interest (Oglat et al., 2018). Although these measurements can be suggested to provide insights into the efficiency of blood circulation and potential improvements resulting from senolytic therapies, these are not routinely utilized tools, particularly in small hospitals or clinics.

The results of this study underscore the significant importance of establishing the safety and efficacy profile of new senolytic drugs. Results from completed trials of D + Q using intermittent dosing (i.e., consecutive treatment for 2–3 days with 2-week resting period) appears to be tolerable, with mild to modest adverse effects (Wissler Gerdes et al., 2021; Orr et al., 2023). Hence, it is crucial to establish the safety profile and dosing regimen of new senolytic drugs to harness the beneficial effects whilst minimizing risks. From the healthcare practitioners’ perspective, it is crucial to ensure that the new senolytic therapy does not interact with other medications, particularly for elderly individuals who are already managing multiple conditions and taking numerous medications. While drug interaction profiles may be available for repurposed drugs, it may not the case for newly developed senolytics. To this end, potential drug-drug interactions, particularly with medications taken by the elderly individuals for their comorbidities have yet to be examined in preclinical and clinical studies and warrant future investigations. In this regard, pharmaceutical experts of this study pointed out the importance of addressing both the selectivity and specificity of senolytics. Improving senolytics selectivity for senescent cells while sparring healthy non-senescent cells is crucial to minimize potential side effects. In addition, designing senolytics to target specific subtypes or subpopulation of senescent cells holds equal importance. Senolytic drugs may target different pro-survival networks within senescent cells to induce cell death depending on the specific cell type. This explains the specific nature of senolytic drugs towards certain cell types as observed with D + Q, navitoclax and fisetin (Zhu et al., 2015; Zhu et al., 2017). Therefore, the strategic development of senolytics with high specificity towards certain cell types such as adipose cells linked to metabolic syndrome and related diseases while preserving the dwindling number of postmitotic cells such as chondrocytes and neurons during aging will yield substantial benefits. The heterogeneous nature of different senescent cell types further presents difficulty in identifying a universal marker that can exclusively target them (Rossi and Abdelmohsen, 2021). Researchers are actively working to identify and validate specific surface markers or molecular signatures that are unique to senescent cells in the vasculature. These biomarkers could serve as targets for developing drug delivery strategies that not only promote both specificity but also enable selective cytotoxicity towards senescent cells. These approaches include natural killer cells (NK)-mediated cytotoxicity, chimeric antigen receptor T (CAR-T) cells, antibody-conjugated nanoparticles, and neutralizing antibodies or soluble receptors (Rossi and Abdelmohsen, 2021). While improving the specificity toward senescent cell of the vasculature remains a challenge, ongoing research and advancements in identifying specific markers and mechanisms associated with senescent cells are likely to contribute to the development of more targeted and effective senolytic therapies for vascular aging in the future. In addition, regulatory agencies such as the Food and Drug Administration (FDA), rely on pivotal trials to provide evidence of the effectiveness and benefit of senolytic therapy. Given the scarcity of pharmacovigilance data on novel senolytic drugs, it is crucial to prioritize further investigation and research in this area. One area of interest is to repurpose FDA approved drugs with good safety profiles such as metformin for screening and identification of novel senolytic drugs (Chen et al., 2022).

The significance of establishing the commercial viability of senolytic therapy has been emphasized by pharmaceutical experts. This study revealed that understanding the market size and competitiveness of senolytic therapy for vascular aging-related disorders is crucial for pharmaceutical companies to determine its commercial viability. Understanding the market size helps pharmaceutical companies gauge the revenue potential and market penetration for their senolytic drugs. In this regard, the market size for senolytic therapy in the context of vascular aging is expected to be substantial. Cardiovascular diseases are a leading cause of morbidity and mortality globally (World Health Organization, 2023). There is a significant demand for an effective therapy that can slow down or reverse the age-related deterioration of blood vessels.

In terms of competitiveness, senolytic therapy must possess the ability to distinguish itself from other competing products in the market. This requires senolytic therapies to possess distinguishing features in terms of efficacy, safety profile, cost-effectiveness, and unique characteristics when compared to alternative treatments or competing products.

The healthcare practitioners in this study also highlight that if senolytic therapy for vascular aging-related disorders becomes available, ensuring its affordability for patients is important. Given the high prevalence of cardiovascular disease among individuals with limited financial resources or low socioeconomic status (Schultz et al., 2018), it is crucial to ensure that the cost of senolytic treatment does not become excessively burdensome for patients. Additionally, healthcare professionals have raised concerns about potential gaps in their knowledge regarding senolytic therapy for vascular aging-related disorders, as it is a relatively new concept that may not have been covered during their medical education. Indeed, the geroscience hypothesis of delaying or treating aging to collectively cure all age-related morbidities still faces unresolved challenges that need to be addressed for its successful implementation (Le Bourg, 2022). Hence, it is crucial to provide education and training to healthcare workers alongside the introduction of senolytic therapy. Additionally, it is vital to establish training programs for healthcare practitioners, equipping them with the necessary skills to effectively communicate and educate patients with lower levels of education about senolytic therapy. The concept of senolytic treatment targeting a fundamental aging mechanism may pose challenges in comprehension for individuals with lower levels of education.

This study has several limitations that should be acknowledged. Firstly, the participants may not fully represent all healthcare workers and pharmaceutical experts, introducing a potential sampling bias. Secondly, all the healthcare workers included in the study were geriatricians, which limits the generalizability of findings to other medical specialties. However, it is important to note that the primary objective of this study was not to compare views among the healthcare practitioners and pharmaceutical experts but rather to gather comprehensive opinions to ensure data saturation and richness.

## 5 Conclusion

Senolytic drugs targeting cellular senescence in the vascular system represents a novel therapeutic strategy with the potential to prevent or delay multiple age-related cardiovascular conditions. However, it is important to highlight a few issues that warrant careful consideration. Researchers and scientists in the field of senolytic therapy may need to establish dosing regimen and address potential outcome-related issues. These include the identification of specific senescence-specific targets, as well as ensuring the safety and efficacy of the therapy with minimal drug-drug interactions. Despite the substantial cardiovascular disease burden and the consequent potentially huge market size, senolytic therapy for vascular aging-related therapy may face competition from existing drugs or supplements targeting the same conditions. Therefore, it is essential to highlight the unique aspects and benefits of senolytic therapy to distinguish it from other approaches. Considering the challenges related to the acceptability of the new senolytic therapy, it is crucial to prioritize maintaining a reasonable cost. The introduction of a new senolytic therapy for vascular senescent cells necessitates the education of the general public and healthcare workers about this innovative treatment. Addressing the knowledge gap is crucial to enhance acceptance and understanding of this therapy.

## Data Availability

The raw data supporting the conclusion of this article will be made available by the authors, without undue reservation.
